# Parents and Children Should Be More Active Together to Address Physical Inactivity and Sedentary Behaviours

**DOI:** 10.3389/fpubh.2022.633111

**Published:** 2022-04-08

**Authors:** Daniel G Rainham, Mark Bennett, Christopher M Blanchard, Sara FL Kirk, Laurene Rehman, Michelle Stone, Daniel Stevens

**Affiliations:** ^1^School of Health and Human Performance, Dalhousie University, Halifax, NS, Canada; ^2^Healthy Populations Institute, Dalhousie University, Halifax, NS, Canada; ^3^Nova Scotia Health, Halifax, NS, Canada; ^4^Department of Medicine, Dalhousie University, Halifax, NS, Canada

**Keywords:** sedentary behaviour, children, adolescents, physical inactivity, intervention

## Abstract

Increasing rates of physical inactivity and sedentary behaviours among children and the youth are important determinants of chronic disease. Supporting children's participation in organised physical activities like sports has been promoted as a public health strategy to increase physical activity. Evidence shows that successful interventions are family-focused, although research on how parental eating and physical activity behaviours influence children's behaviours is deficient. In this commentary, we argue that interventions for countering physical inactivity and sedentary behaviours should include greater focus on home and social environments, specifically the influence and involvement of parents, siblings, and friends in supporting these health behaviours. We conclude that the design of interventions to prevent chronic diseases in children should also consider more carefully the conditions in which the behaviours of children and their parents occur. This means encouraging parents and children to be active together to address physical inactivity and sedentary behaviours, while being mindful of unintended consequences of focusing on one behaviour over another.

## Introduction

Participation in regular and sustained physical activity has important health benefits, such as improved cardiovascular, bone, and mental health (1, 2). However, in Canada, only around 39% of 5–17 year-olds meet the physical activity recommendations in the 24-h Movement Guidelines for Children and Youth (1, 2). Recent data also reveal that Canadian children (6–11 years) spend 7.5 to 7.9 h per day, or 62% of waking hours, engaged in sedentary behaviours (3, 4). Alongside physical inactivity and sedentary behaviours, healthy eating is also important. Dietary intakes of children and the youth are suboptimal, with intake of energy dense, nutrient-poor ultra-processed foods making up more than 55% of total daily energy intake among children and adolescents (5). Together, lack of movement and poor dietary quality are important determinants of chronic diseases and their risk factors such as youth obesity; about one-third of Canada's population between 6 and 17 years of age (31.4%) are overweight or obese (6), a tripling of prevalence over the last 30 years (7). Public health actions to prevent obesity and chronic disease among children and the youth, therefore, need to recognise factors that influence these important health behaviours, whilst being mindful of broader socio-ecological determinants that may perpetuate bias and stigma (8). In this commentary, we discuss why interventions to promote physical activity should include greater focus on home and social environments, specifically the influence and involvement of parents, siblings, and friends, while avoiding unintended consequences that might arise by focusing on one health behaviour over another.

## Paradox of Privileging One Health Behaviour Over Another

Promoting physical activity is remarkably more complicated when there is misalignment among key health-promoting behaviours, and when there are unintended consequences arising from intentions to support them. In other words, interventions that typically focus on one behaviour, like physical activity or healthy eating, may inadvertently ignore the influence or impact of other behaviours. For example, detailed GPS-derived location data coupled with actimetry and nutritional quality surveys from more than 360 children and youth (ages 8–15 years) revealed that: (1) in individuals, physical activity behaviour is not necessarily associated with healthy eating behaviour (i.e., children who were more physically active did not eat well); (2) children studied were largely sedentary but were more physically active when actively commuting (walking to school), during organised sports or after school activities, or when they were with friends (e.g., at a mall); and (3) a major barrier to healthy eating was the time constraint arising from familial commitments to organised sports and related recreational activities (9–11). Paradoxically, scheduling of activities to promote physical activity outside of school was of consequence for dietary quality, which was sacrificed because of time pressures associated with leisure time and organised sport activities. Of note, a recent narrative review of factors influencing children's eating behaviours (12) does not mention the role of physical activity on familial eating behaviours, suggesting that there is limited research in this area (11). Furthermore, determinants of health outcomes associated with physical activity and sedentary behaviours are not necessarily analogous. Although youth who participate in sport are more likely to be physically active, they are also more likely to consume greater amounts of calories and some unhealthy foods and beverages (13). What these data tell us is that we need to be mindful of the unintended consequences of privileging one health behaviour (e.g., physical activity) over another (e.g., healthy eating).

Children and adults may also meet physical activity guidelines but stay sedentary for extended periods of time throughout the day. For example, during the school year, children may sit for a long time in formal classroom settings with only short breaks for recess and meals. Children may also engage in formal or organised physical activities, such as training in sport activities, and then return home only to resume sedentary behaviours such as watching television or other screen-related activities. A major challenge for physical activity interventions, then, is the potential influence of home and social environments, and, specifically how parents, siblings, and friends might exert influence on health behaviours. Opportunities to intervene in sedentary behaviours are context-specific (i.e., school, home, and commuting by car) and not necessarily amenable to activity substitution. Rather than focus on introducing more physical activity, such as participation in sports or family-oriented physical activities, interventions focused on posture change or modification of sedentary behaviours may also lead to demonstrable health benefits (14).

## Family Matters for Health Promotion

Interventions are also more effective when they move beyond focus on individual children and youth, and incorporate broader influences of other family members or friends. There are several important reasons not to overlook the role of parents and siblings, as well as friends, in older children. Parents can assist in applying tailored behavioural change strategies that are sensitive to the familial, developmental, and social contexts of their children. For example, parents can have considerable influence on sedentary behaviours, particularly by limiting their own sedentary behaviours and using screens or other technologies that promote sedentary behaviours at home (15). Although familial influence on health-promoting behaviours declines as children age (2), there is evidence of value in promoting the importance of physical activity to parents, especially to fathers, and encouraging them to increase their physical activity in order to increase the physical activity levels of low-active children and adolescents (16). Children are also more likely to be physically active when their parents promote unstructured active play (17), including participation in nature-based recreation and spending more time outdoors, which also have important mental health benefits (18). Parents can further support, reinforce, and model both positive and negative nutrition and activity behaviours (19). Some interventions to promote physical activity or reduce sedentary behaviours are family-focused, and there are limited high-quality data on the effectiveness of intervention designs that are centred on parental involvement (20).

Of the interventions that exist, effective ones tend to involve both parents and children in the implementation of intervention activities, principally by increasing familial physical activity levels, as well as targeting of food choices and appropriate behaviour change skills (21). For example, a review on parental perceptions of healthy child behaviours found that family environment and intergenerational influences are highly influential on weight-related behaviours, including sharing of knowledge, attitudes, and beliefs around nutrition and physical activity (22). Family-focused interventions early in child development are also more likely to be effective than those later in childhood or adolescence in the development of healthy nutrition and activity behaviours.

**Figure 1:**
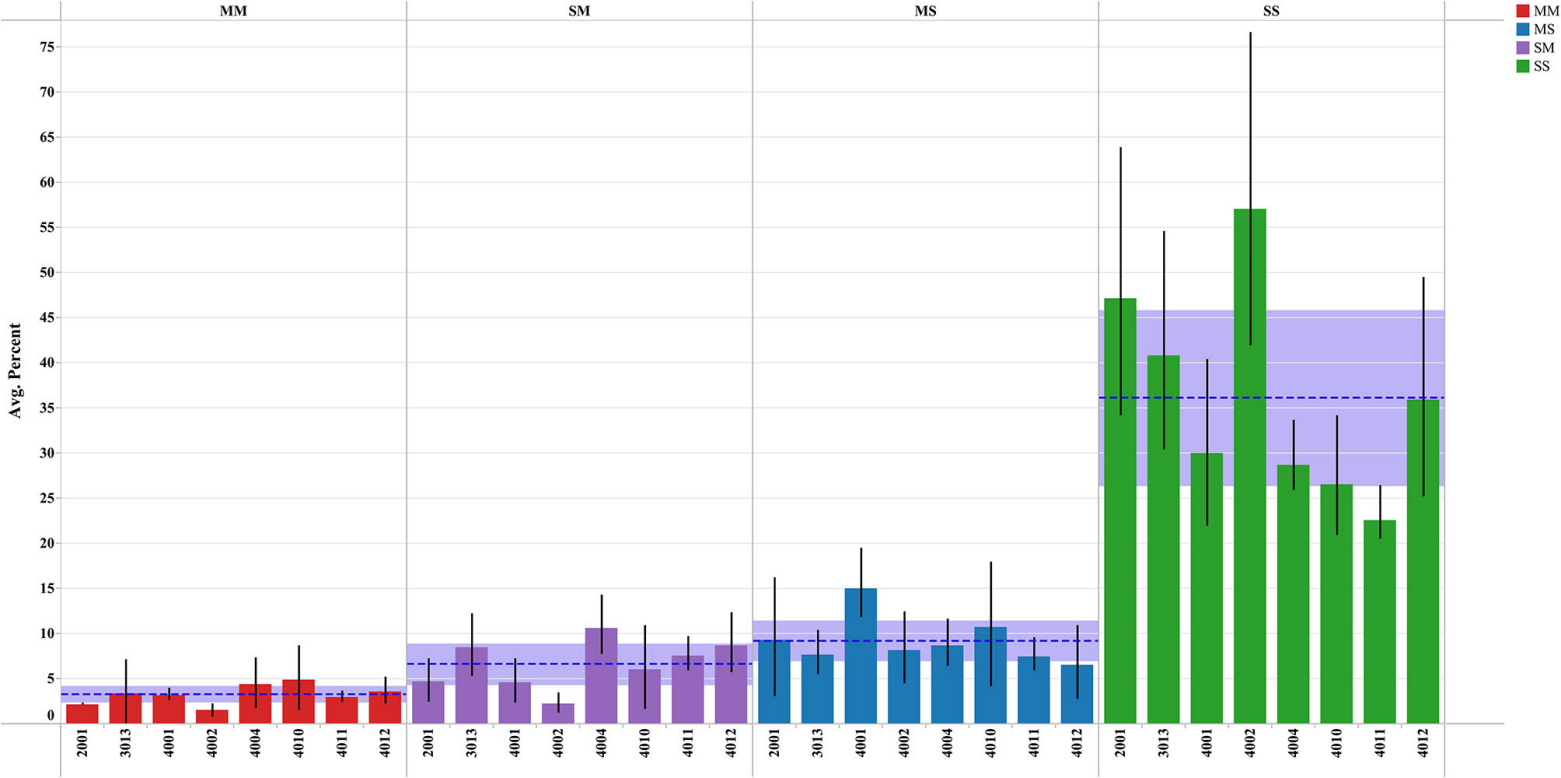
Percent of time children and parents spend together (within 50 m) in moderate to vigorous physical activity and sedentary time. MM, both engaged in MVPA; SM, child is sedentary and parent in MVPA; MS, child in MVPA and parent is sedentary; SS, both child and parent are sedentary. Results are limited to data with following restrictions: nine or more measurement pair hours per day; participants with more than four valid days of accelerometer wear time; and participants (parent-child pairs) within a 50 m range of each other.

Remarkably, we know very little about the influence of intrafamilial behaviours, for example how parental eating and physical activity behaviours influence children's behaviours (and vice versa). Results from qualitative inquiry and self-report data indicate that families rarely participate in physical activities together (23). Larger parent-child studies on joined activity behaviour based on objectively measured data (i.e., data from accelerometry and global positioning systems) reported that 10–16% of moderate to vigorous physical activity and 41–46% of sedentary behaviours occurred together, with the majority of joint sedentary behaviours occurring at home (24, 25). Our own preliminary results, based on objective measurement data from eight parent-child pairs, show that, on average, 4% of parent-child moderate-to-vigorous physical activity (MVPA) and more than 35% of sedentary behaviours occur together. Time spent in MVPA, by either a child or a parent, increased slightly when only the parent or the child was conducting MVPA in the same location (Figure 1). A similar pattern has been noted for mother-child pairs where the majority of shared time was spent engaged in sedentary or light-activity behaviours (26). More recent investigations of families as the unit of analysis have implemented cluster-based analyses to identify family-level health behavioural typologies (healthy, unhealthy, and divergent) associated with physical activity and eating behaviours (27). Interventions focused on modifying activity behaviours of children should also consider actions to reduce family-level sedentary behaviours. Not surprisingly, the data support the view that we may need to design interventions that reduce sedentary behaviours rather than fixating on only the promotion of more physical activities.

## Conclusion

There is little disagreement that well-designed and properly funded interventions are required to promote healthy behaviours among children. A common thread and challenge to preventing chronic disease in children through physical activity promotion is acknowledgement, consideration, and integration of context (place and time) into interventions. Interventions are more likely to succeed if they also target conditions in which human behaviours occur.

A fruitful context of focus for intervening to support health behaviours in children is the family, particularly parents and siblings. Surprisingly, only few interventions involve parents and siblings, even though they model health behaviours and can directly influence sedentary behaviours. It may be unwise to ignore the larger societal context in which families operate. We could all agree that there are many individual and societal barriers to the adoption of health promoting behaviours; logically, these barriers are more likely to be surmounted as a family or group than as an individual. A solution is for parents to be more active with their children.

## Data Availability Statement

The raw data supporting the conclusions of this article will be made available by the authors, without undue reservation.

## Ethics Statement

The studies involving human participants were reviewed and approved by Dalhousie University Research Ethics Board. Written informed consent to participate in this study was provided by the participants' legal guardian/next of kin.

## Author Contributions

DR and SK conceived the research and study design, planned the acquisition, analysis, and interpretation of the data. MB made substantial contributions in the analysis, interpretation of the data, and revision of the manuscript. DR was accountable for all aspects of the study and responsible for ensuring the accuracy and integrity of the study. All authors participated in data collection, preliminary analysis, and early drafting of the manuscript and made substantive contributions to the development and revision of themanuscript. All authors contributed to the article and approved the submitted version.

## Funding

This research was supported by the Canadian Institutes of Health Research; Institute of Human Development, Child and Youth Health; Institute of Nutrition, Metabolism and Diabetes; and the Heart and Stroke Foundation of Canada through the Built Environment, Obesity and Health Initiative.

## Conflict of Interest

MB was employed by Nova Scotia Health, Halifax, NS, Canada. The remaining authors declare that the research was conducted in the absence of any commercial or financial relationships that could be construed as a potential conflict of interest.

## Publisher's Note

All claims expressed in this article are solely those of the authors and do not necessarily represent those of their affiliated organizations, or those of the publisher, the editors and the reviewers. Any product that may be evaluated in this article, or claim that may be made by its manufacturer, is not guaranteed or endorsed by the publisher.

## References

[1] Canadian, Society for Exercise Physiology (CSEP),. Canadian 24-h Movement Guidelines for Children and Youth (Ages 5–17 Years). CSEP. SCPE. Available online at: https://csepguidelines.ca (accessed June 28, 2021).

[2] PartcipACTION. The Role of the Family in the Physical Activity, Sedentary and Sleep Behaviours of Children and Youth. The 2020 ParticipACTION Report Card on Physical Activity for Children and Youth. Toronto, ON: ParticipACTION (2020).

[3] Colley RC Garriguet D Janssen I Craig CL Clarke J Tremblay MS. Physical activity of Canadian children and youth: accelerometer results from the 2007 to 2009 Canadian Health Measures Survey. Health Rep. (2011) 22:15–23. 21510586

[4] Prince SA Melvin A Roberts KC Butler GP Thompson W. Sedentary behaviour surveillance in Canada: trends, challenges and lessons learned. Int J Behav Nutr Phys Act. (2020) 17:34. 10.1186/s12966-020-00925-8 32151285 PMC7063715

[5] Moubarac J-C Batal M Louzada ML Martinez Steele E Monteiro CA. Consumption of ultra-processed foods predicts diet quality in Canada. Appetite. (2017) 108:512–20. 10.1016/j.appet.2016.11.006 27825941

[6] Rao DP Kropac E Do MT Roberts KC Jayaraman GC. Childhood overweight and obesity trends in Canada. Chronic Dis Injuries. (2016) 36:194–8. 10.24095/hpcdp.36.9.03 27670922 PMC5129778

[7] Janssen I. The public health burden of obesity in Canada. Can J Diabet. (2013) 37:90–6. 10.1016/j.jcjd.2013.02.059 24070798

[8] Salas XR. The ineffectiveness and unintended consequences of the public health war on obesity. Can J Public Health. (2015) 106:E79. 10.17269/cjph.106.4757 25955676

[9] Rainham DG Bates CJ Blanchard CM Dummer TJ Kirk SF Shearer CL. Spatial classification of youth physical activity patterns. Am J Prev Med. (2012) 42:e87–96. 10.1016/j.amepre.2012.02.011 22516507

[10] Shearer C Blanchard C Kirk S Lyons R Dummer T Pitter R. Physical activity and nutrition among youth in rural, suburban and urban neighbourhood types. Can J Public Health. (2012) 103:S55–60. 10.1007/BF03403836 23618091 PMC6973923

[11] Chircop A Shearer C Pitter R Sim M Rehman L Flannery M. Privileging physical activity over healthy eating: ‘Time’ to Choose? Health Promot Int. (2015) 30:418–26. 10.1093/heapro/dat056 23945086 PMC4542915

[12] Scaglioni S De Cosmi V Ciappolino V Parazzini F Brambilla P Agostoni C. Factors influencing children's eating behaviours. Nutrients. (2018) 10:706. 10.3390/nu10060706 29857549 PMC6024598

[13] Nelson TF Stovitz SD Thomas M LaVoi NM Bauer KW Neumark-Sztainer D. Do youth sports prevent pediatric obesity? A systematic review and commentary. Curr Sports Med Rep. (2011) 10:360–70. 10.1249/JSR.0b013e318237bf74 22071397 PMC4444042

[14] Spence JC Rhodes RE Carson V. Challenging the dual-hinge approach to intervening on sedentary behavior. Am J Prev Med. (2017) 52:403–6. 10.1016/j.amepre.2016.10.019 27939240

[15] Lindsay AC Sussner KM Kim J Gortmaker S. The role of parents in preventing childhood obesity. Future Child. (2006) 16:169–86. 10.1353/foc.2006.0006 16532663

[16] Edwardson CL Gorely T. Parental influences on different types and intensities of physical activity in youth: a systematic review. Psychol Sport Exerc. (2010) 11:522–35. 10.1016/j.psychsport.2010.05.001

[17] Appelhans BM Li H. Organized sports and unstructured active play as physical activity sources in children from low-income Chicago households. Pediatr Exerc Sci. (2016) 28:381–7. 10.1123/pes.2015-0249 26757032

[18] Lovelock B Walters T Jellum C Thompson-Carr A. The participation of children, adolescents, and young adults in nature-based recreation. Leisure Sci. (2016) 38:441–60. 10.1080/01490400.2016.1151388

[19] Beets MW Cardinal BJ Alderman BL. Parental social support and the physical activity-related behaviors of youth: a review. Health Educ Behav. (2010) 37:621–44. 10.1177/1090198110363884 20729347

[20] Müller MJ Danielzik S Pust S. School- and family-based interventions to prevent overweight in children. Proc Nutr Soc. (2005) 64:249–54. 10.1079/PNS2005424 15960869

[21] Golley RK Hendrie GA Slater A Corsini N. Interventions that involve parents to improve children's weight-related nutrition intake and activity patterns – what nutrition and activity targets and behaviour change techniques are associated with intervention effectiveness? Obesity Rev. (2011) 12:114–30. 10.1111/j.1467-789X.2010.00745.x 20406416

[22] Pocock M Trivedi D Wills W Bunn F Magnusson J. Parental perceptions regarding healthy behaviours for preventing overweight and obesity in young children: a systematic review of qualitative studies. Obesity Rev. (2010) 11:338–53. 10.1111/j.1467-789X.2009.00648.x 19780989

[23] Thompson JL Jago R Brockman R Cartwright K Page AS Fox KR. Physically active families – de-bunking the myth? A qualitative study of family participation in physical activity. Child Care Health Dev. (2010) 36:265–74. 10.1111/j.1365-2214.2009.01051.x 20047594

[24] Dunton GF Kawabata K Intille S Wolch J Pentz MA. Assessing the social and physical contexts of children's leisure-time physical activity: an ecological momentary assessment study. Am J Health Promot. (2012) 26:135–42. 10.4278/ajhp.100211-QUAN-43 22208410

[25] Dunton GF Liao Y Almanza E Jerrett M Spruijt-Metz D Pentz MA. Locations of joint physical activity in parent–child pairs based on accelerometer and GPS monitoring. Ann Behav Med. (2013) 45:162–72. 10.1007/s12160-012-9417-y 23011914 PMC3562385

[26] Dlugonski D DuBose KD Rider P. Accelerometer-measured patterns of shared physical activity among mother–young child dyads. J Phys Activity Health. (2017) 14:808–14. 10.1123/jpah.2017-0028 28556667

[27] Niermann CYN Spengler S Gubbels JS. Physical activity, screen time, and dietary intake in families: a cluster-analysis with mother-father-child triads. Front Public Health. (2018) 6:276. 10.3389/fpubh.2018.00276 30324100 PMC6172305

